# Genetic Variation Analysis and Development of KASP Marker for Leaf Area and Hight in Southern-Type *Populus deltoides*

**DOI:** 10.3390/plants14030330

**Published:** 2025-01-23

**Authors:** Chunxiao Liu, Jiawei Yan, Zhongxu Zhang, Lu Pei, Caihua Li, Xiaoman Zhang, Shengqing Shi

**Affiliations:** 1 College of Landscape Architecture and Tourism, Hebei Agricultural University, Baoding 071001, China; liuchunxiao@pgs.hebau.edu.cn (C.L.); zhangzhongxu@pgs.hebau.edu.cn (Z.Z.); 2 State Key Laboratory of Tree Genetics and Breeding, Key Laboratory of Tree Breeding and Cultivation of State Forestry and Grassland Administration, Research Institute of Forestry, The Chinese Academy of Forestry, 1958 Box, Beijing 100091, China; yanjiawei@caf.ac.cn (J.Y.); pl2219947120@163.com (L.P.); 3 Shijiazhuang Academy of Agriculture and Forestry Sciences, Shijiazhuang 050041, China; licaihua415@126.com

**Keywords:** *Populus deltoides*, GWAS, phenotypic traits, KASP markers

## Abstract

*Populus deltoides* holds significant ecological and economic importance and is a crucial gene donor for the world’s staple poplar varieties. To select and breed *P. deltoides* with improved agronomic traits, nine growth and leaf traits were examined in 375 different genotypes, assessing their genetic diversity and performing correlation and comprehensive ranking analyses. Phenotyping results were then utilized to screen a total of 2,009,263 SNP (single nucleotide polymorphism) loci significantly associated with the nine phenotypic traits. A total of 45 SNP loci exhibited significant associations with growth traits based on a general linear model (GLM) analysis. By analyzing the Linkage disequilibrium (LD) block of five SNP loci with significant leaf area and height, we identified five candidate genes related to leaf area and height. Three of the five SNP loci were successfully validated using KASP (kompetitive allele-specific PCR) assays. One loci Chr08_16007979 was closely linked with leaf area, and two loci Chr05_12148738, and Chr05_17106547 were closely linked with height. The developed functional KASP markers offer valuable insights for subsequent further marker-assisted breeding and genetic improvement studies in southern-type poplars.

## 1. Introduction

*Populus deltoides* W. Bartram ex Marshall can grow up to 50 m in height and is characterized by rapid growth and stress resistance. The stem is straight and complete, with moderate branch thickness and relatively high smoothness [1]. It is widely used in forestry, environmental protection, landscaping, ecological engineering, and wood production [2,3]. The study of the genetic diversity of germplasm resources is crucial for identifying superior *P. deltoides* germplasm, varieties, or major traits [4]. As a fundamental method for studying the genetic diversity of germplasm resources, phenotypic identification is widely utilized in germplasm resource characterization [5,6].

There are currently many reports on the phenotypic traits of poplar trees [7,8,9]. For example, environmental resource utilization traits and growth traits of *P. deltoides* have been analyzed [10,11], leading to the selection of the clones with the best overall performance. Chen [12] analyzed 384 individuals from six regions with 15 pairs of simple sequence repeat markers, which revealed that the southern-type *P. deltoides* exhibited a high degree of differentiation, consistent with the phenotypic trait analyses by Cao [13], Chen [14], and Yan [15], which explored *P. deltoides* diversity in growth and leaf traits. These phenotypic trait studies have laid a foundation for the subsequent selection of superior *P. deltoides* clones.

Molecular breeding encompasses marker-assisted selection (MAS) and whole genome selection [16]. The construction of genetic and physical maps using various marker types helps to identify important quantitative trait loci (QTLs) or the underlying candidate genes [17] for MAS [18,19]. High-density molecular markers can also be used for genome-wide association studies (GWAS) [20,21]. With the development of high-throughput sequencing, numerous recent studies have been conducted on the phenotypic and genetic characterization of poplar genotypes and the mining of related SNP loci through GWAS. These include correlations of stress tolerance with poplar leaf phenotypes, mining key genes, and investigating the genetic structure of leaf shape variation [22,23,24,25,26,27]. Single nucleotide polymorphisms (SNPs) are the most common genetic markers in genome-wide association studies and are usually in linkage disequilibrium (LD) with each other within a small genomic region [28]. Currently, GWAS [29] and KASP [30] are being widely applied in dissecting the genetic regulatory mechanisms of complex plant traits. GWAS is a powerful tool for resolving complex quantitative traits [31,32]. Fahrenkrog [33] conducted the first genome-wide association analysis in *P. deltoides*. Our understanding of the genetic mechanisms underlying leaf shape variation in poplar has been enhanced by GWAS analysis of poplar leaf shape [18,34,35,36,37]. The genetic characterization of traits is also assessed by GWAS analysis of poplar phenotypes, which provides candidate genes for breeding. Kompetitive allele-specific PCR (KASP) is widely used in the identification of cytoplasmic fertility, molecular marker-assisted breeding, identification of germplasm resources, and transgene detection [38,39,40]. Its advantages of easy operation, low cost, high throughput and high accuracy [41,42,43], can be successfully applied to a variety of plants to provide the basis for efficient molecular breeding.

Here, we measured the growth traits (height and diameter at breast height) and leaf indices of 375 germplasm individuals from a Southern-type *P. deltoides* germplasm bank. The genetic diversity of 375 *P. deltoides* genotypes was analyzed and evaluated comprehensively. GWAS were performed, and KASP markers were developed. This work provides candidate markers linked to key agronomic traits for molecular breeding.

## 2. Results

### 2.1. Variation Analysis of Phenotypic Data

Cluster analysis revealed nine growth and leaf phenotypic traits that were distributed across 10 classes (Supplementary Figure S1). Leaf fresh weight (FW) was primarily distributed in classes 3–7, representing 84.27% of the germplasm resources. Leaf dry weight (DW) and length-to-width ratio were mainly distributed in classes 4–7, accounting for 79.57% and 86.13% of the germplasm resources, respectively. Leaf area, petiole length, leaf water content, specific leaf area (SLA), diameter at breast height (DBH), and height (H) were predominantly distributed in classes 3–8, comprising 89.08%, 87.47%, 87.47%, 86.13%, 86.4%, and 86.67% of the genotypes assessed, respectively. These findings may indicate significant variation in the nine traits among different *P. deltoides* genotypes.

**Figure 1:**
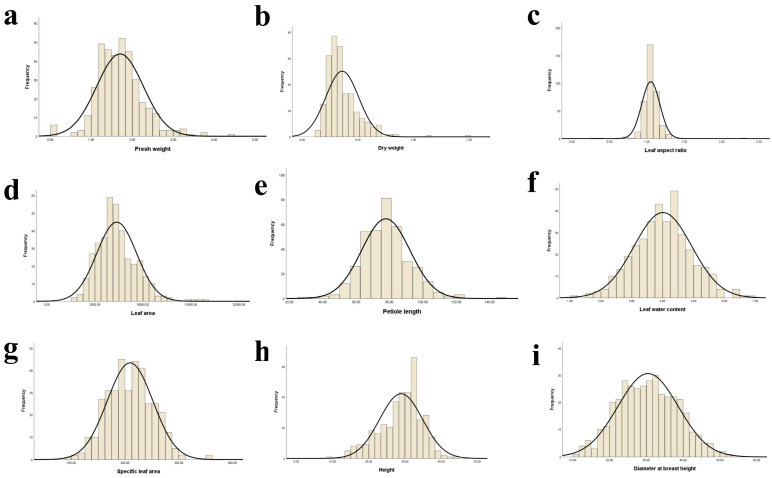
Distribution frequency of traits in *Populus deltoides* germplasm resources. (**a**–**i**): fresh weight (g), dry weight (g), leaf aspect ratio, leaf area (mm^2^), petiole length (mm), leaf water content, specific leaf area (g/mm^2^), height (m), diameter at breast height (cm).

The variations in phenotypic traits were analyzed in the 375 genotypes (Table 1). The diversity indices of the nine traits ranged from 1.66 to 2.10, with coefficient of variation (CV) values from 11.4% to 41.7% and an average value of 24.59%. Leaf dry weight, leaf fresh weight, and leaf area exhibited significant variation, as evidenced by their high coefficients of variation. Conversely, the lowest coefficients of variation observed in leaf aspect ratio, petiole length, and SLA suggest that these three traits exhibited less variation and more stable genetic characteristics. The H-value of the genetic diversity index ranged from 1.66 to 2.10, with all nine traits exhibiting values greater than 1. Tree height, DBH, SLA, leaf water content, and petiole length exhibited greater diversity indices, suggesting a more uniform distribution of these traits across the germplasm. In contrast, in the diversity indices for length-to-breadth ratio, DW, and FW were lower, suggesting uneven distribution among phenotypes. The assessed *P. deltoides* traits followed a normal or near-normal distribution (Figure 1), making them suitable for genome-wide association studies (GWAS).

**Figure 2:**
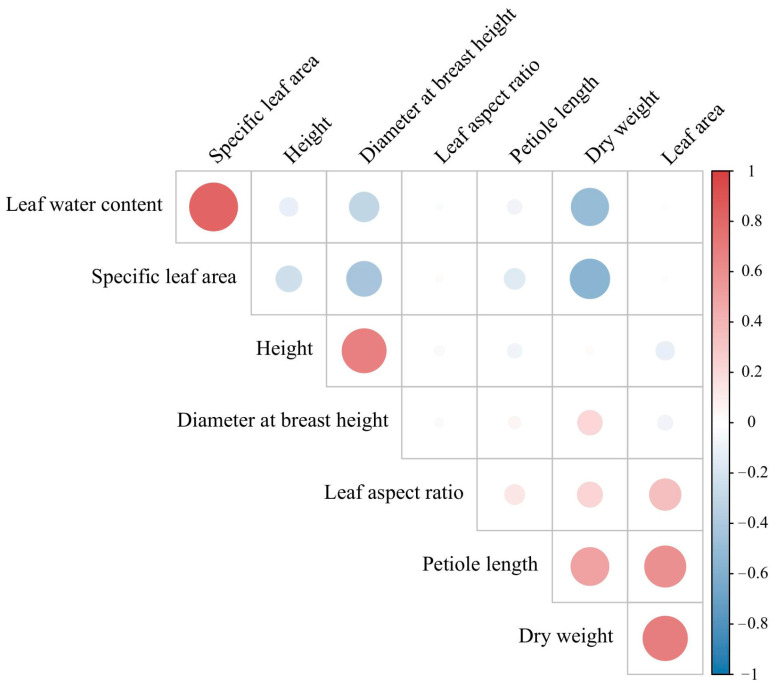
Trait correlations in the 375 *Populus deltoides* germplasm collection. Red circles indicate a positive correlation; a larger circle size denotes a higher correlation. Blue circles indicate a negative correlation; larger circle size denotes a lower correlation.

The correlation analysis of traits in 375 *P. deltoides* germplasm resources revealed the following significant relationships (Figure 2): FW was significantly positively correlated with the DW, leaf aspect ratio, leaf area, and petiole length (*p* < 0.01), and significantly negatively correlated with the specific leaf area (*p* < 0.01). DW was positively correlated with the leaf aspect ratio, leaf area, and petiole length (*p* < 0.01) and negatively correlated with leaf water content and SLA (*p*< 0.01). The leaf aspect ratio was positively correlated with petiole length (*p* < 0.01). Leaf area was positively correlated with petiole length (*p* < 0.01) and negatively correlated with H (*p* < 0.05). Leaf water content was positively correlated with SLA (*p* < 0.01) and negatively correlated with H (*p* < 0.05) and DBH (*p* < 0.01). SLA was negatively correlated (*p* < 0.01) with H and DBH. Tree height was positively correlated (*p* < 0.01) with DBH. Significant (*p* < 0.05) or highly significant (*p* < 0.01) correlations were identified among the traits across the poplar germplasm population.

The 375 southern-type *P. deltoides* genotypes originated from different geographic locations, categorized into 13 different regions. The regional germplasm resource trends were as follows: genotypes from the Greenville area had the highest leaf FW and DW mean values; genotypes from the Texas A&M area had the highest SLA values; genotypes from the Washington area had the highest leaf water content values for and H and DBH mean values; genotypes from the Southern Forestry Experiment Station had the highest leaf area mean values. There were significant differences (*p* < 0.05) in all six traits across regions except for DW, LWR, and H. *P. deltoides* genotypes from Issaquena had significantly lower FW and leaf area compared to other regions. Similarly, *P. deltoides* genotypes from the Southern Forestry Experiment Station had the lowest water content, while those from Sandbar 8 had the highest chest diameter. Additionally, *P. deltoides* from the Hempstead area had the highest SLA mean values (Supplementary Table S1).

The growth environments of *P. deltoides* can be divided into several categories. Clusters in the same category share similar characteristics, while clusters from different categories exhibit more distinct differences. K-means clustering, an unsupervised classification method, provides high accuracy, high intraclass similarity, and high significant interclass differentiation [44]. In this study, we employed k-means clustering to determine the final number of clusters and analyze the results. As shown in Supplementary Table S2, the number of clusters in the three categories was 162, 131, and 71, respectively. Category 1 included the highest water content, which is an important index to characterize the response of plant leaves to the environment [45], responding to the water status of the plant. Category 2 included the lowest FW, DW, petiole length, and leaf area but higher DBH and tree height, which are more related to the wood output. Category 3 has the highest FW, DW, and leaf area but the lowest DBH, containing fewer clusters.

Cluster analysis aids in comprehending the kinship and genetic background among resources, facilitating their utilization. Notably, the above three groups lacked distinct geographical clustering traits; however, each group exhibited unique characteristics.

### 2.2. Comprehensive Evaluation of P. deltoides Phenotypic Traits

Principal component analysis of the nine traits (Supplementary Table S3) revealed that the first three principal components accounted for 77.1% of the cumulative variance, indicating that they encapsulate most of the phenotypic variation of the *P. deltoides* germplasm collection.

The first principal component accounted for 37.42% of the variance, with the dominant eigenvector being DW, signifying that it primarily represented a trait associated with leaf weight. The second principal component contributed 25.88%, and the third principal component contributed 13.79%, with the predominant eigenvectors being SLA and H, respectively. Based on the principal component analysis results, DW, SLA, and water content were the main factors contributing to the differences in *P. deltoides* germplasm resources.

The weight coefficients of the three principal components (0.372, 0.256, and 0.138) were used to calculate the composite score (F value) for each genotype. Each genotype was comprehensively evaluated based on its F value, with a higher F value indicating better phenotypic composite traits. The F values of the asexual lines ranged from 0.119 to 0.593, with an average F value of 0.291. Table 2 ranks the combined phenotypic values and lists the top and bottom ten *P. deltoides* genotypes and their regions of origin. Thirty percent of the top ten genotypes with higher composite values were from the Joseph area, and another 30% were from Greenville. On the other hand, 30% of the bottom ten genotypes with the lowest composite values originated from Sandbar 8. The *P. deltoides,* genotype 6–25 from Joseph, had the highest F-value of 0.593, while genotype 304-25 from the Southern Forestry Experiment Station had the lowest F-value of 0.16. Correlation analyses between the nine phenotypic traits and the phenotypic composite values (F-values) (Supplementary Table S4) revealed a significant negative correlation between the F-values and H (*p* < 0.05). Except for a non-significant correlation with DBH, the F-values exhibited highly significant positive correlations (*p* < 0.01) with the remaining seven traits.

### 2.3. GWAS Analysis

**Figure 3:**
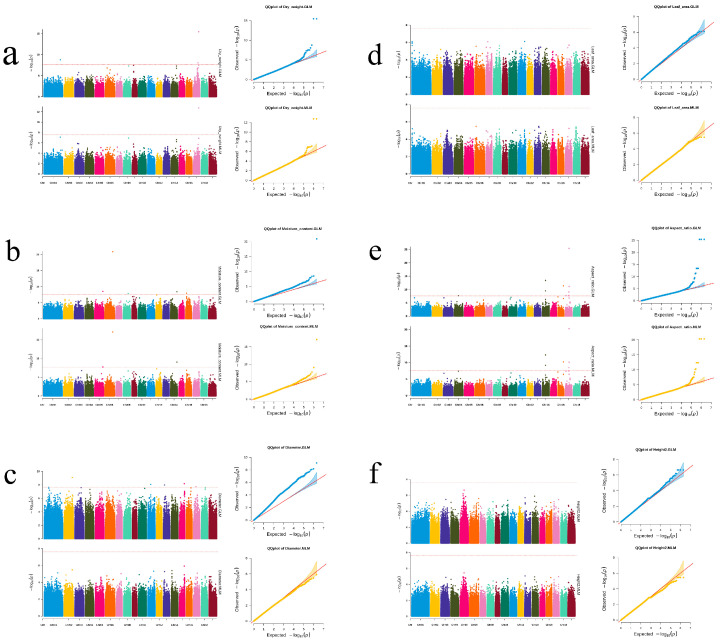
Manhattan and Q-Q plots of the GWAS analysis of *P. deltoides* phenotypes. (**a**–**f**): DW, leaf water content, DBH, leaf area, aspect ratio, H. Above is the Q-Q plot for GML; Below is the Q-Q plot for MLM.

To identify functional SNP loci associated with *P. deltoides* growth and leaf traits, we performed a GWAS analysis on nine phenotypic traits and 2,009,263 high-confidence SNP loci (For gene SNP chip construction, unpublished) of 375 *P. deltoides* germplasms in the GLM and MLM model. The GLM model identified the majority of the associated loci, whereas the MLM model identified a smaller number of associated SNP loci. All loci identified in one model were also identified in the other (Figure 3). The trait-SNP loci associations were positive, as indicated by the Q-Q plots (Figure 3). In total, 45 significantly associated SNP loci were detected: 4 for DW, 13 for mean leaf aspect ratio, 14 for leaf area, 5 for leaf water content, 2 for H, and 7 for DBH (Supplementary Table S5).

**Figure 4:**
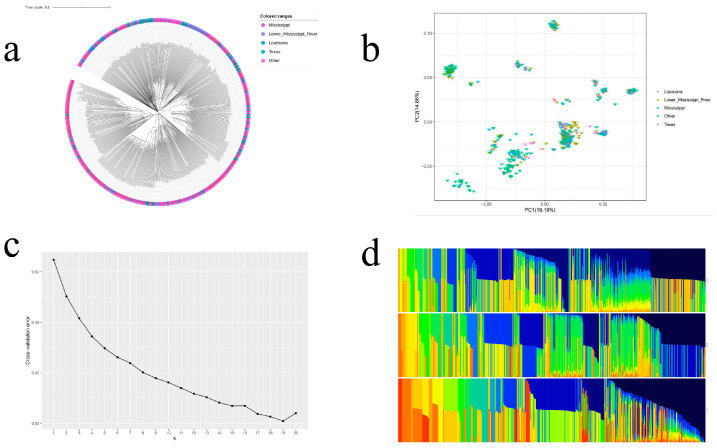
Genetic structure and phylogeny of populations. (**a**) Phylogenetic tree of the *P. deltoides*. The phylogenetic tree was constructed using Phylip with the Maximum likelihood (ML) method. Bootstrap values in percentages (1000 replicates) are indicated on the nodes. (**b**) Scatter plot of 375 *P. deltoides* PCA. (**c**) Trends in K based on admixture software analysis. (**d**) Population genetic structure of *P. deltoides* based on K = 14, 15, 16. Each color represents a different ancestral cluster, each individual is represented as a thin vertical bar.

Cluster analysis and principal component analysis of *P. deltoides* based on 2,009,263 high-quality SNP loci showed that there were obvious clusters in the group, but each cluster contained samples from different geographic regions (Figure 4a,b) The structure analysis of the *P. deltoides* group was plotted on the basis of the presumed K value (Figure 4c,d), and the region of decreasing value of the CV was flat when K = 15. Therefore, *P. deltoides* can be divided into 15 subgroups. Within the 15 subgroups, the majority of individual grouping information is still mixed, and only a few individuals with obvious grouping information are partly hybridized from two ancestral subgroups, indicating that *P. deltoides* populations do not have obvious genetic stratification characteristics.

**Figure 5:**
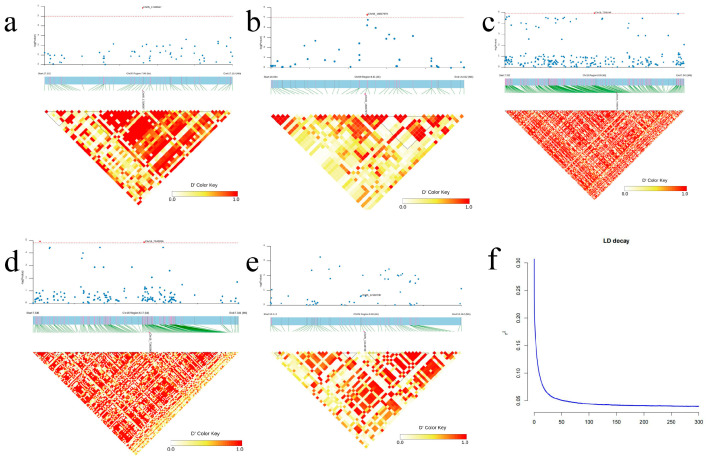
LD block analysis of 5 extremely significant SNP sites. (**a**–**e**): Chr05-17106547, Chr08-16007979, Chr18-7336146, Chr18-7340296, Chr05-12148738. The pairwise linkage disequilibrium (LD) between SNP markers is indicated by the D’ value, where red indicates a value of 1 and yellow indicates a value of 0. Dark red indicates the highest correlation between two SNP with the highest LD. D’ is the normalized linkage disequilibrium coefficient. (**f**): *P. deltoides* population LD decay map.

Analysis of the 8.9 kb region upstream and downstream of five SNP loci significantly associated with leaf area (Chr08-16007979, Chr18-7336146, Chr18-7340296) and H (Chr05-17106547 and Chr05-12148738) using the LD haplotype block diagram based on the LD decay distance ($r^{2}=0.1$), revealed that the five significant SNP sites formed five blocks (Figure 5).

Using the genome of *P. deltoides* as a reference, 5 key genes, including phosphatases, transcription factors, and phosphatase 2C, were identified within 8.9 kb of five SNP loci associated with leaf area and plant height (Table 3). Annotation of the five SNPs showed that none of them were located within the gene. The genes H0E87_016253, H0E87_029602 and H0E87_029603, encoding phosphatase 2C, phosphatidylinositol-3,4,5-trisphosphate 3-phosphatase activity, and regulator of Vps4 activity in the MVB pathway, respectively, functioned on leaf area trait, and the other two H0E87_010606 and H0E87_010813, encoding on transcription factor and receptor-like protein kinase, respectively, are related growth of height.

### 2.4. KASP Markers Development

**Figure 6:**
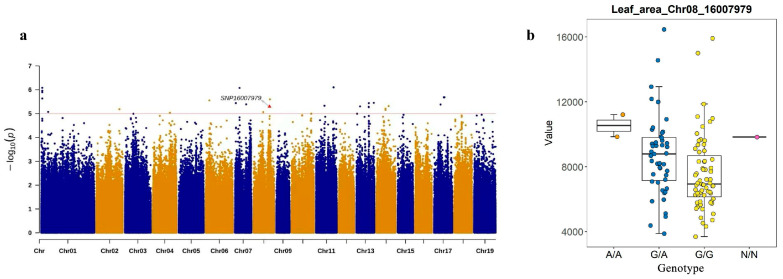
Loci associated with leaf area loci and corresponding boxplots identified by KASP markers analyses in the *P. deltoides* population. (**a**) Manhattan map of KASP markers SNP loci: SNP16007979. (**b**) Pure A allele genotypes were in orange; pure G allele genotypes were in yellow; heterozygous fractions were in blue, unclassified genotype N/N was in pink.

The association between three of the five aforementioned SNPs and leaf area and height traits was validated by analyzing re-sequencing data and evaluating the phenotypic performance of the population. At the Chro8_16007979 loci, the GA genotype was associated with higher leaf area, while the GG genotype was associated with lower leaf area. The polymorphic KASP markers were used to assess the associations of genetic variants with leaf area variation in the *P. deltoides* population. A highly informative locus, Chr08_16007979 (Figure 6), was selected to classify a subset of genotypes consisting of 96 *P. deltoides* germplasms. Of these, 95 contained two A/A genotypes, 45 genotypes were G/A, 50 genotypes were G/G, and one genotype could not be classified.

**Figure 7:**
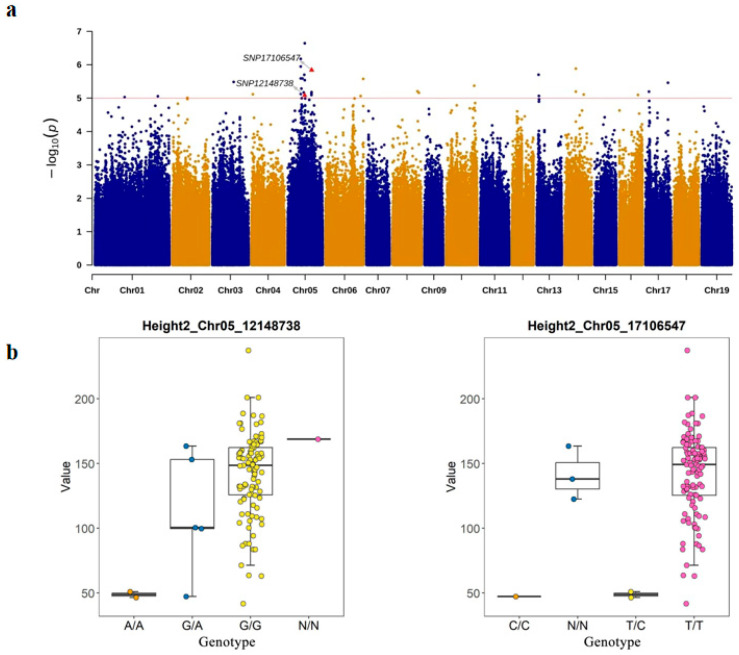
Loci associated with plant height and corresponding boxplots identified by KASP marker analyses in the *P. deltoides* population. (**a**) Manhattan map of KASP markers SNP loci: SNP12148738, SNP17106547. (**b**) Left panel: Pure A allele genotypes were in orange; pure G allele genotypes were in yellow; heterozygous fractions were in blue. Right panel: pure C allele genotype was in orange; pure T allele genotypes were in pink; heterozygous fractions were in yellow; unclassified genotypes N/N were in blue.

Similarly, at the Chr05-12148738 loci, the GG genotype was associated with high growth of height, whereas the GA genotype was associated with low growth of height; at the Chr05-17106547 loci, the TT genotype was associated with high growth of height, while the TC genotype was associated with low growth of height. KASP marker analysis was used to genotype the population H, and two loci were screened to classify a population of 96 *P. deltoides* genotypes. Locus Chr05_12148738 distinguished two individuals with genotype A/A, five individuals with genotype G/A, and one unclassified genotype. Locus Chr05_17106547 distinguished one individual with genotype C/C, two individuals with genotype T/C, and three unclassified genotypes (Figure 7).

## 3. Discussion

Genetic diversity provides the foundation for evaluating and utilizing germplasm, offering essential insights for the exploitation of genetic resources. Phenotypic traits serve as a key basis for analyzing complex physiological and growth mechanisms in *P. deltoides* [46,47,48,49]. The study revealed variations in the genetic diversity among phenotypic traits in the different regions. *P. deltoides* germplasm resources exhibited a high mean diversity index of 1.96 for phenotypic and growth traits, with the highest diversity index observed for DBH (2.1), indicating high genetic diversity and a broad genetic basis [50]. Correlation analysis revealed that leaf DW and FW were significantly and positively correlated with most other traits, suggesting that increased leaf biomass may improve properties and metrics related to other traits. SLA was significantly negatively correlated with both H and DBH. Asexually propagated lines with lower SLA may have advantages in aboveground biomass growth, positioning them as a prospective target in future breeding efforts to enhance the value of *P. deltoides* in timber production. *P. deltoides* genotypes from different geographic origins exhibited distinct characteristics, which can be used to select parental materials aligned with desired breeding objectives and strategies. This provides a reference value for discovering favorable genes in the germplasm and their exploitation, enabling superior species improvement and directed breeding strategies.

The affiliation function value method, correlation, and principal component analysis were employed in this study to comprehensively evaluate *P. deltoides*, taking into account its phenotypic traits [51]. Leaf-related traits had a greater contribution to the first principal component, similar to the results of Hao Lei’s study on the phenotypic diversity of *Salix psammophila* germplasm resource populations [52]. The overall quality of each clone was assessed using the comprehensive score (F value) of phenotypic traits. The combined evaluation showed that all the other traits were significantly positively correlated except for H and DBH, which were negatively correlated with the composite value. Therefore, the higher the composite value, the higher the corresponding value of these traits. Among the top ten and bottom ten genotypes regarding their composite values, 30% of the top ten were from Joseph, 30% were from Greenville, while 30% of the bottom ten were from Sandbar 8. This serves as a reference for breeding efforts and the investigation of regional resources for optimal variety selection in Hubei Province. The experimental results obtained in this study under only one well-cultivated condition have some limitations, and the experimental results may differ from this study in other environments.

A limited number of loci may control complex growth traits in forest trees [53]. In this study, five key genes were screened from 8.9 kb upstream and downstream of the five important SNP loci (Figure 4, Table 3). Previous studies have shown that the underlying genes may be involved in cell signaling and development functions [54,55]. The candidate gene H0E87_010606 associated with Chr05_12148738 is located on chromosome 5 and homologous to *Arabidopsis thaliana* encodes a Protein kinase superfamily protein, Protein kinases are major players in various signaling pathways and may play a crucial role in plant responses to adverse environments [56]. The candidate gene H0E87_016253 associated with Chr08_16007979 is located on chromosome 8 and homologous to *Arabidopsis thaliana* encoding PP2C5, a member of the PP2C family phosphatases. Phosphorylation and dephosphorylation, regulated by protein kinases and protein phosphatases, respectively, are considered to be key signal transduction mechanisms in plant responses to the environment. PP2C5 acts as a MAPK phosphatase that positively regulates seed germination, stomatal closure and ABA-inducible gene expression. An important role of PP2C is the regulation of biotic and abiotic stress responses, potassium (K) deficiency signaling, plant immunity and development [57,58]. The two candidate genes screened in this experiment may be related to the differences in the performance of different individuals of *P. deltoides* under natural growth conditions, and thus can be used as candidate genes for further functional validation.

Analysis of the results of the population structure, *P. deltoides* populations are not clearly characterized by genetic stratification, a phenomenon that may indicate the following: (1). Gene flow and migration: There may have been a gene exchange migratory event between *P. deltoides* in different geographic regions that resulted in their sharing certain genetic characteristics, which suggests that gene flow between these populations is not uncommon [59,60]. (2). Environmental adaptations: Although poplars from different geographic regions have specific adaptations, they may also share some basic adaptive traits, which makes them show some similarity in PCA analysis. (3). The distribution of genetic variation: Poplars from different geographic regions may have a relatively wide range of genetic variation, therefore, on the first and second principal components of PCA, these variants may not be completely differentiated by geographic region.

Competitive allele-specific PCR (KASP) is a widely recognized technique for SNP genotyping [61]. KASP has been extensively used in various plant breeding applications, such as identification of germplasm resources, quality control (QC) analysis, allele mining, linkage localization, quantitative trait loci (QTL) localization, genetic mapping, development of trait-specific markers, and MAS [62]. It has been successfully implemented in *Oryza sativa*, *Triticum aestivum*, *Amygdalus persica*, *Vitis vinifera*, and other plants [63,64,65,66]. In this study, we identified three KASP marker loci: Chr08_16007979, Chr05_12148738, and Chr05_17106547, which were linked to the leaf area and H, respectively. The three KASP markers verified the validity of the functional genes above, suggesting their potential as selection markers for growth traits. In future research, we will verify the function of the candidate genes and explore their expression patterns and molecular regulatory mechanisms through molecular biology techniques. This will provide a theoretical foundation regarding gene regulation underlying the complex growth traits of *P. deltoides*.

## 4. Materials and Methods

### 4.1. Overview of the Trial Site

Located in Dongsheng Town, Shishou City, Jingzhou City, Hubei Province, it is situated at the intersection of the Jianghan Plain and the Dongting Lake Plain, characterized by a subtropical monsoon climate. It receives sufficient solar radiation, abundant heat, and a long frost-free period. The annual sunshine hours are 1800–2000 h, the average annual temperature is 15.9–16.6 °C, the annual cumulative temperature is ≥10 °C, the annual frost-free period is 242–263 d, and the rainfall in most years is between 1100 and 1300 mm.

### 4.2. Plant Materials

The *P. deltoides* Marshall asexual forest was planted in 2011, with a row spacing of 4 m × 6 m, totaling 799 *P. deltoides* asexual lines, with the position of each asexual line being completely randomized. The 799 asexual lines were from Greenville, Wcoahowa, Southern Forestry Experiment Station, Issaquena, Texas A&M, other regions, Wbolivar, Sandbar 8, Sandbar 29, Washington, Hempstead, and Brunswick. A total of 375 asexual lines of *P. deltoides* Marshall were randomly selected as materials, and the diameter at breast height (DBH) and height (H) of each asexual line were measured. One plant from each line was selected for leaf collection, and its leaf phenotypic traits were measured.

### 4.3. Phenotypic Trait Determination

Diameter at breast height (DBH) was measured in 2023 for *P. deltoides* in the forest with a breast diameter ruler (accurate to 0.1 cm), and height (H) was measured by using a Vertex IV ultrasonic altimeter (accurate to 0.01 m, Haglof, Långsele, Sweden). The leaf collection time was in the middle and late May. Between 6 and20 mature leaves were randomly collected in the middle and lower periphery of the canopy of each clonal line to be used for analysis. The collected leaves were randomly divided into three replicates, with each replicate containing no fewer than two leaves. The fresh weight of the leaves was weighed with an electronic balance (accuracy of 0.01 g). Then, the leaf petiole length, leaf area, and leaf aspect ratio were measured quickly. After the measurement was completed, the leaves were placed in an electric blast drying oven (101-0DB, Taisite, Tianjin, China) and dried at 95 °C until they reached a constant weight.

### 4.4. Phenotypic Trait Analysis

The trait classes were based on the mean ($\bar{x_{i}}$) and standard deviation (δ) of each trait, and the traits were categorized into 1–10 classes according to $\bar{x_{i}}\pm k\delta$ (where k = 0, 0.5, 1, 1.5, 2).

Distribution frequency: $P_{ij}=\frac{n_{ij}}{n},\;P_{ij}$ denotes the frequency of distribution of the *j*th variant of the *i*th trait, $n_{ij}$ denotes the number of genotypes in the *j*th variant of the *i*th trait, and *n* denotes the total number of genotypes.

The coefficient of variation (*CV*) indicates the degree of dispersion of a trait: CV=δ/μ (δ is the standard deviation, μ is the average value).

Shannon-Weaver index of genetic diversity (*H*):

$$H=-\sum_{n=1}^{n}P_{ij}\ln(P_{ij})$$

### 4.5. Comprehensive Evaluation of Phenotypic Traits

Microsoft Excel 2017 was used to process data on 9 phenotypic traits of 375 *P. deltoides* germplasm accessions. Each principal component score and the combined score of the clonal lines were calculated by principal component analysis for comprehensive evaluation. The standardized values of the 9 traits of the asexual lines were utilized and were incorporated into the three principal components mentioned above, resulting in the following linear equations for the three principal components:

First principal component:$Y_{1}=0.51X_{1}+0.46X_{2}+0.45X_{3}+0.38X_{4}+0.18X_{5}+0.13X_{6}-0.28X_{7}-0.24X_{8}+0.03X_{9}$

Second principal component: $Y_{1}=-0.05X_{1}+0.23X_{2}+0.30X_{3}+0.16X_{4}+0.15X_{5}-0.48X_{6}+0.47X_{7}+0.43X_{8}-0.41X_{9}$

Third principal component: $Y_{1}=-0.12X_{1}+0.17X_{2}+0.13X_{3}+0.09X_{4}+0.10X_{5}+0.446X_{6}+0.33X_{7}+0.47X_{8}+0.62X_{9}$

The ANOVA, correlation analysis, and principal component analysis were conducted using the SPSS 19.0 statistical software.

### 4.6. GWAS and KASP Assay

Whole genome resequencing at 20× was conducted on 375 *P. deltoides*, yielding a total of 5.243 terabases of raw data. Following the filtration of low-quality data from the downstream raw data using FASTP, the total sequencing clean data for all samples amounted to 4.997 terabases. A summary of the sequencing data output and quality for the first 10 samples is detailed in Supplementary Table S6. For the hard-filtered SNP data after GATK, the final filtering was performed using VCF tools (--max-missing 0.9 --maf 0.05 --max-alleles 2 ---min-alleles 2), retaining only SNPs with SNP genotype detection rate of 90% or above, MAF of 0.05 or above, and dimorphic SNPs. Filtering resulted in 2,009,263 high-confidence SNP loci. Reference genome: https://www.ncbi.nlm.nih.gov/datasets/genome/GCA_015852605.2/ (accessed on 20 December 2021).

Utilizing the rMVP package in R. Two models, GLM and MLM, were employed to assess the association of 2,009,263 high-confidence SNP loci with the phenotypic data of nine growth traits. A −log⁡10 (p>7.5) significance threshold was used to filter the significant SNP association loci. Finally, Manhattan plots and Quantile-Quantile (Q-Q) scatter plots were plotted in R language (Version: 4.1.1). LD decay: PopLDdecay Version: 3.4, LD Block: LD BlockShow Version: 2.6.

A minimum of 150 bp of flanking sequences both upstream and downstream of the marker site were extracted, and the sequences obtained were aligned with the reference genome to eliminate multi-copy sequences, high GC content, and highly repetitive sequences. The Batchprimer 3 software was used to design KASP primers for SNP sites and flanking sequences. Each set of KASP markers consisted of two specific primers and one universal primer, with fluorescent junction sequences attached to the 5′ end of the specific primers (GAAGGTGACCAAGTTCATGCT was the FAM fluorescent junction sequence; GAAGGTCGGGAGTCAAC GGATT was the HEX fluorescent connector sequence). KASP primer sequences are detailed in Supplementary Table S7. KASP marker validation was performed in 96 samples on a Douglas Scientific Array Tape system. The PCR system was assembled by NEXAR and is shown in the Supplementary Table S8. After the PCR reaction, the reaction system was scanned for fluorescence signals by ARAYA, and then the data were analyzed and genotyped by INTELLICS.

## 5. Conclusions

In this study, phenotypic trait evaluation was performed on 375 *P. deltoides* genotypes, leading to the identification of superior genotypes and providing technical support for resource evaluation and exploitation. GWAS of growth-related traits revealed a total of 45 loci associated with phenotypes, along with three KASP markers closely linked to leaf area and H. This study offers significant insights for the identification and utilization of advantageous genes in *P. deltoides* germplasm resources, establishing a foundation for comprehending the regulation of intricate growth traits. Further investigation into functional loci associated with growth is necessary to deepen our understanding of growth regulation mechanisms in *P. deltoides*. These findings provide a scientific basis for optimizing *P. deltoides* growth traits and serve as a reference for molecular-assisted breeding focused on key phenotypic traits. This will accelerate molecular breeding progress, increase the number of improved *P. deltoides* varieties, and significantly contribute to the efficient and sustainable development of poplar breeding.

## Figures and Tables

**Table 1:** Distribution characteristics of 9 traits in 375 *Populus deltoides* germplasm resources.

Character	Minim	Maximum	Median	Average	SD	CV	H
Fresh weight (g)	0.62	4.44	1.67	1.73	0.53	0.31	1.94
Dry weight (g)	0.14	1.50	0.33	0.36	0.15	0.42	1.66
Leaf aspect ratio	0.30	2.25	1.05	1.05	0.12	0.11	1.73
Leaf area (mm^2^)	2754.41	16,466.43	7007.38	7260.93	2074.31	0.29	2.00
Petiole length (mm)	27.90	144.44	77.04	77.74	14.43	0.19	2.03
Water content of leaves	1.06	6.99	4.00	4.01	0.93	0.23	2.04
Specific leaf area (g/mm^2)^	97.89	357.71	210.34	209.25	42.29	0.20	2.05
Height (m)	9.50	44.00	29.90	28.94	5.89	0.20	2.04
Diameter at breast height (cm)	10.50	52.20	30.29	30.45	8.15	0.27	2.10

**Table 2:** Ranking of *Populus deltoides* phenotypes and phenotypic composite values by the region of origin.

Genotypes	Area	F	Genotypes	Area	F
6-25	Joseph	0.593	373-27	Issaquena	0.180
S3105-4	Greenville	0.590	814-18	Sandbar 8	0.178
329-26	Sandbar 29	0.568	503-28	Greenville	0.171
94-4	Brunswick	0.488	S3814-12	Sandbar 8	0.166
S3127-27	Southern Forestry Experiment Station	0.482	323-9	Wbolivar	0.165
302-12	Southern Forestry Experiment Station	0.475	313-14	Wcoahowa	0.163
S3264-11	Greenville	0.464	14-2	Sandbar 8	0.160
S3264-3	Greenville	0.451	304-25	Southern Forestry Experiment Station	0.150
65-18	Joseph	0.442	305-11	Southern Forestry Experiment Station	0.145
6-9	Joseph	0.437	324-10	Wbolivar	0.119

The left column represents the ten genotypes with the highest ranked F-values; the right column represents the ten genotypes with the lowest ranked F-values.

**Table 3:** List of candidate genes.

Linkage SNP Loci	Ref	Alt	Gene Name	Gene Start	Gene End	Function
Chr05_12148738	G	A	H0E87_010606	12153189	12154992	transcription factor
Chr05_17106547	A	T	H0E87_010813	17139852	17141451	receptor-like protein kinase
Chr08_16007979	A	G	H0E87_016253	16051537	160453578	phosphatase 2C
Chr18_7336146	C	T	H0E87_029602	7373639	7375145	phosphatidylinositol-3,4,5-trisphosphate 3-phosphatase activity
Chr18_7340296	G	A	H0E87_029603	7382800	7387358	Regulator of Vps4 activity in the MVB pathway

## Data Availability

Data is contained within the article or Supplementary Materials.

## Supplementary Materials

The following supporting information can be downloaded at: https://www.mdpi.com/article/10.3390/plants14030330/s1.
