# Supplementary material for: Genetic Variation Analysis and Development of KASP Marker for Leaf Area and Hight in Southern-Type Populus deltoides
Source: Plants (Basel). 2025 Jan 23;14(3):330. doi: 10.3390/plants14030330 (PMC11820701; doi:10.3390/plants14030330)
Supplement: Supplementary file 1 [file plants-14-00330-s001.zip › plants-3386406-supplementary.pdf]

Supplementary Table S1. Differences and variance analysis of 9 phenotypic traits of populus clones in different geographical locations

Area	DW	FW	Leaf aspect ratio	SLA	Petiole length	Water content of leaves	Leaf area	H	DBH
Greenville	1.96±0.81ab	0.42±0.19	1.08±0.07	208.43±38.13ab	81.93±14.53ab	3.86±0.99abc	8483.10±2638.46a	29.25±5.56	29.01±7.38abc
Wcoahowa	1.78±0.46abc	0.39±0.13	1.05±0.10	207.48±46.40ab	80.55±11.96b	3.82±1.05bc	7781.65±1973.29abc	29.19±5.74	31.15±8.31abc
Southern Forestry Experiment Station	1.83±0.51abc	0.41±0.15	1.05±0.08	203.56±45.47ab	81.63±15.05ab	3.68±0.90c	8050.25±2205.14abc	28.45±5.17	28.90±7.47abc
Issaquena	1.50±0.36c	0.31±0.13	1.03±0.06	217.19±45.58a	72.96±11.68bc	4.16±0.87	6390.05±1399.93c	30.25±5.62	30.83±9.31abc
Texas A&M	1.71±0.45abc	0.33±0.12	1.07±0.07	234.09±39.78a	75.76±11.63bc	4.42±1.02abc	7533.41±2216.86abc	27.41±6.52	27.94±6.77bc
Other regions	1.76±0.54abc	0.38±0.20	1.06±0.07	201.31±39.18ab	78.18±12.20b	4.02±0.89abc	7094.44±1875.73	28.76±6.46	31.79±9.59abc
Wbolivar	1.52±0.45c	0.31±0.09	1.05±0.09	224.17±43.01a	75.25±14.22bc	4.08±0.83abc	6898.70±1938.39abc	27.92±6.46	30.30±7.79abc
Sandbar 8	1.73±0.49abc	0.38±0.14	1.05±0.08	182.15±25.99b	76.56±14.31b	3.71±0.74bc	6791.28±1920.60bc	30.80±6.03	35.03±7.35a
Sandbar 29	1.64±0.48abc	0.34±0.13	1.04±0.11	211.19±41.04ab	80.27±10.70b	4.08±0.98abc	6989.29±1979.77abc	31.09±5.68	33.07±7.08ab
Washington	1.57±0.31bc	0.30±0.08	1.06±0.05	214.60±42.00ab	65.03±14.60c	4.48±1.18ab	6530.29±1097.97c	32.31±3.62	35.36±6.96a
Hempstead	1.71±0.55abc	0.32±0.15	1.05±0.07	226.19±30.42a	75.17±11.18bc	4.40±0.61abc	7129.64±1630.69abc	25.96±4.02	26.56±8.41bc
Brunswick	2.07±0.44a	0.37±0.07	1.09±0.06	220.22±45.71a	78.71±7.25b	4.60±0.96a	8029.55±1451.29abc	27.71±4.60	26.16±6.33c

Supplementary Table S2. Clustering results and characteristic parameters

Category	FW	DW	Leaf aspect ratio	Leaf area	Petiole length	Leaf water content	SLA	PH	DBH	Clusters
Category 1	1.78	0.36	1.05	7468.76	79.51	4.11	212.18	28.22	30.42	162
Category 2	1.27	0.27	1.04	5330.84	69.76	3.96	206.43	29.91	31.14	131
Category 3	2.44	0.53	1.10	10499.08	89.70	3.85	205.00	28.54	29.27	71

Supplementary Table S3. Feature vectors, eigenvalues, contribution rates and cumulative contribution rates of the three principal components of 9 phenotypic traits

	PC1	PC2	PC3
FW	0.51	-0.05	-0.12
DW	0.46	0.23	0.17
Leaf area	0.45	0.30	0.13
Petiole length	0.38	0.16	0.09
Leaf aspect ratio	0.18	0.15	0.10
DBH	0.13	-0.48	0.46
SLA	-0.28	0.47	0.33
Water content of leaves	-0.24	0.43	0.47
H	0.03	-0.41	0.62
Characteristic value	3.37	2.33	1.24
Contribution rate（%）	37.42	25.88	13.79
Cumulative contribution（%）	37.42	63.31	77.10

Supplementary Table S4. Correlation coefficients between 9 phenotypic traits and phenotypic composite value (F-value)

Phenotypic	F-value
DW	.874**
FW	.577**
Leaf aspect ratio	.470**
Leaf area	.911**
Petiole length	.683**
Water content of leaves	.267**
SLA	.200**
H	-.119*
DBH	-0.101

** At the 0.01 level, the correlation is significant. * At the 0.05 level, the correlation is significant.

Supplementary Table S5 Details of 45 SNP loci

Trait	SNP	CHROM	POS	REF	ALT	Effect	SE	GLM
DW	Chr01-46524500	Chr01	46524500	A	G	-0.4079	0.06603	1.72E-09
DW	Chr17-13163884	Chr17	13163884	A	C	0.150142	0.025508	8.87E-09
DW	Chr17-15066464	Chr17	15066464	A	C	-1.17809	0.137914	3.48E-16
DW	Chr17-15066480	Chr17	15066480	T	A	-1.17809	0.137914	3.48E-16
Leaf aspect ratio	Chr04-11682956	Chr04	11682956	T	G	-0.65595	0.114387	2.04E-08
Leaf aspect ratio	Chr14-9077244	Chr14	9077244	A	G	-0.62944	0.080063	4.23E-14
Leaf aspect ratio	Chr14-9077247	Chr14	9077247	A	G	-0.62944	0.080063	4.23E-14
Leaf aspect ratio	Chr14-9077270	Chr14	9077270	C	T	-0.43219	0.066905	3.32E-10
Leaf aspect ratio	Chr16-15417170	Chr16	15417170	A	G	-0.46318	0.064602	4.15E-12
Leaf aspect ratio	Chr17-1874020	Chr17	1874020	A	G	0.655947	0.114387	2.04E-08
Leaf aspect ratio	Chr17-9734417	Chr17	9734417	T	C	-1.18282	0.103405	3.95E-26
Leaf aspect ratio	Chr17-9734429	Chr17	9734429	C	T	-0.38	0.065861	1.69E-08
Leaf aspect ratio	Chr17-9734455	Chr17	9734455	C	T	-0.37969	0.066341	2.17E-08
Leaf aspect ratio	Chr17-9743860	Chr17	9743860	A	G	-0.41189	0.065369	8.45E-10
Leaf aspect ratio	Chr17-10383540	Chr17	10383540	C	T	-1.18282	0.103405	3.95E-26
Leaf aspect ratio	Chr17-10383559	Chr17	10383559	T	C	-0.56399	0.079521	6.80E-12
Leaf aspect ratio	Chr17-10383907	Chr17	10383907	G	A	1.182819	0.103405	3.95E-26
Leaf area	Chr08-16007979	Chr08	16007979	A	G	0.18316	0.137914	1.79E-13
Leaf area	Chr14-9077210	Chr14	9077210	A	C	-0.62944	0.080063	4.23E-14
Leaf area	Chr14-9077244	Chr14	9077244	A	G	-0.62944	0.080063	4.23E-14
Leaf area	Chr14-9077247	Chr14	9077247	A	G	-0.62944	0.080063	4.23E-14
Leaf area	Chr14-9077270	Chr14	9077270	C	T	-0.43219	0.066905	3.32E-10
Leaf area	Chr16-15417170	Chr16	15417170	A	G	-0.46318	0.064602	4.15E-12
Leaf area	Chr17-1874020	Chr17	1874020	A	G	0.655947	0.114387	2.04E-08
Leaf area	Chr17-9734417	Chr17	9734417	T	C	-1.18282	0.103405	3.95E-26
Leaf area	Chr17-9734429	Chr17	9734429	C	T	-0.38	0.065861	1.69E-08
Leaf area	Chr17-9734455	Chr17	9734455	C	T	-0.37969	0.066341	2.17E-08
Leaf area	Chr17-9743860	Chr17	9743860	A	G	-0.41189	0.065369	8.45E-10
Leaf area	Chr17-10383540	Chr17	10383540	C	T	-1.18282	0.103405	3.95E-26
Leaf area	Chr18-7336146	Chr18	7336146	C	T	-0.56399	0.079521	6.80E-12
Leaf area	Chr18-7340296	Chr18	7340296	G	A	1.182819	0.103405	3.95E-26
Leaf water content	Chr05-23129207	Chr05	23129207	T	G	-4.60688	0.757986	3.08E-09
Leaf water content	Chr06-22216000	Chr06	22216000	C	A	-10.064	0.98765	1.28E-21
Leaf water content	Chr08-14537056	Chr08	14537056	G	T	-4.45243	0.766477	1.37E-08
Leaf water content	Chr14-18006452	Chr14	18006452	C	T	3.242095	0.537067	3.88E-09
Leaf water content	Chr16-2134103	Chr16	2134103	T	C	1.035389	0.176925	1.08E-08
H	Chr05_12148738	Chr05	12148738	G	A	-3.48827	0.59489	7.94E-09
H	Chr05_17106547	Chr05	17106547	T	C	-1.18563	0.104407	3.89E-26
DBH	Chr01-13259073	Chr01	13259073	C	G	-3.70678	0.652914	2.24E-08
DBH	Chr02-23011442	Chr02	23011442	C	T	-3.39352	0.54248	8.11E-10
DBH	Chr11-8329750	Chr11	8329750	T	A	-3.79926	0.64961	8.63E-09

DBH	Chr13-3216319	Chr13	3216319	A	T	-3.50439	0.602891	1.06E-08
DBH	Chr15-14042624	Chr15	14042624	T	C	2.954141	0.501689	6.89E-09
DBH	Chr17-6664822	Chr17	6664822	A	G	-5.52795	0.96838	1.89E-08
DBH	Chr18-10818974	Chr18	10818974	C	T	-4.2875	0.757152	2.43E-08

Supplementary Table S6 A summary table of the quality of data output

Samples	Rawreads	Rawbases	Cleanreads	Cleanbases	Cleanrate	Clean Q20	CleanQ 30	Depth	GC_rate
P1	73,240,942	10,986,141,300	72,043,778	10,645,908,690	96.90%	98.84%	95.78%	10.65	37.67%
P2	57,048,656	8,557,298,400	56,481,166	8,386,093,257	98.00%	98.85%	95.78%	8.39	36.86%
P3	72,812,262	10,921,839,300	71,656,910	10,611,188,993	97.16%	98.80%	95.65%	10.61	37.03%
P4	60,745,928	9,111,889,200	60,025,092	8,911,447,471	97.80%	98.73%	95.39%	8.91	36.85%
P5	65,178,700	9,776,805,000	64,485,696	9,582,288,016	98.01%	98.76%	95.49%	9.58	36.79%
P6	59,332,802	8,899,920,300	58,626,254	8,707,562,173	97.84%	98.61%	95.00%	8.71	36.71%
P7	65,322,492	9,798,373,800	64,450,956	9,562,112,446	97.59%	98.75%	95.47%	9.56	36.54%
P8	61,755,556	9,263,333,400	60,861,276	9,021,092,287	97.38%	98.75%	95.45%	9.02	36.74%
P9	59,950,678	8,992,601,700	59,126,282	8,769,443,718	97.52%	98.79%	95.59%	8.77	36.93%
P10	55,724,998	8,358,749,700	54,982,830	8,155,951,982	97.57%	98.72%	95.38%	8.16	37.65%

Rawreads: total number of raw reads; Rawbases: total number of raw bases; Cleanreads: total number of filtered reads; Cleanbases: total number of filtered bases; Cleanrate: ratio of clean bases/raw bases; CleanQ20: percentage of bases with quality value ≧ 20; CleanQ30: percentage of bases with quality value ≧ 30; Depth: sequencing depth, clean bases/GenomeSize; GC_rate: percentage of GC-content bases in sequencing data.

Supplementary Table S7 KASP primer sequences

		Allele
Chr01_46524500	F1:GAAGGTGACCAAGTTCATGCTCAAGTTAAGTGATTCAAAACTCGCA**A**	A
	F2:GAAGGTCGGAGTCAACGGATTCAAGTTAAGTGATTCAAAACTCGCA**G**	G
	R:GGCCAATATCTAGCTGTGGGTAGTTA	
Chr01_46524500	F1:GAAGGTGACCAAGTTCATGCTCCAAGTTAAGTGATTCAAAACTCGCA**A**	A
	F2:GAAGGTCGGAGTCAACGGATTCCAAGTTAAGTGATTCAAAACTCGCA**G**	G
	R:TTGTGGCCAATATCTAGCTGTGGGTA	
Chr17_4303180	F1:GAAGGTGACCAAGTTCATGCTTCTCAGAATCTTGGATTCGAACTCAAA**T**	T
	F2:GAAGGTCGGAGTCAACGGATTCTCAGAATCTTGGATTCGAACTCAAA**C**	C
	R:CCCCATGCTCACAAAGAAACCTCTAT	
Chr03_10629367	F1:GAAGGTGACCAAGTTCATGCTAGCCACAGGCAGCAAAGAAGTT**T**	T
	F2:GAAGGTCGGAGTCAACGGATTAGCCACAGGCAGCAAAGAAGT**C**	C
	R:CAATTAAACCTCTCAGTTCCGTGACCA	
Chr13_4868909	F1:GAAGGTGACCAAGTTCATGCTCCAAGACTGATTTGGAATGCTTAGGTTATA**G**	G
	F2:GAAGGTCGGAGTCAACGGATTCCAAGACTGATTTGGAATGCTTAGGTTATA**T**	T
	R:TCAGCGACGCATCAGACTACCTAACT	
Chr13_3216319	F1:GAAGGTGACCAAGTTCATGCTGAAAAACACGAATGAGATTCACATCT**T**	A

	F2:GAAGGTCGGAGTCAACGGATTTGAAAAACACGAATGAGATTCACATCT**A**	T
	R:GTCAAAGATGCGAGTTTTGTATTTGA	
Chr15_14042624	F1:GAAGGTGACCAAGTTCATGCTGTCAGAAATTGGCTCTTACAAGGAA**A**	T
	F2:GAAGGTCGGAGTCAACGGATTGTCAGAAATTGGCTCTTACAAGGAA**G**	C
	R:TCCAACTTAATCGAGGATTCTAATTTTTC	
Chr08_16007979	F1:GAAGGTGACCAAGTTCATGCTATGGCAGTTCAGGGAAGCTC**A**	A
	F2:GAAGGTCGGAGTCAACGGATTATGGCAGTTCAGGGAAGCTC**G**	G
	R:GGAAGGAGATATGGATGGCCCAGTAT	
Chr18_7336146	F1:GAAGGTGACCAAGTTCATGCTCACTTTTGCCCCCTTGTAAGAG**C**	C
	F2:GAAGGTCGGAGTCAACGGATTCTCACTTTTGCCCCCTTGTAAGAG**T**	T
	R:CTTGAGAAAATTAACTTTGGCTAAGATCA	
Chr18_7340296	F1:GAAGGTGACCAAGTTCATGCTCATCTGTTGATAGAAGCCAGCTCTT**G**	G
	F2:GAAGGTCGGAGTCAACGGATTGCATCTGTTGATAGAAGCCAGCTCTT**A**	A
	R:CCCATGTCATCTCCATATAGGACACG	
Chr04_11682956	F1:GAAGGTGACCAAGTTCATGCTCAAGTCGGTTCAGTGAGGGC**T**	T
	F2:GAAGGTCGGAGTCAACGGATTAGTCGGTTCAGTGAGGGC**G**	G
	R:CTTGATTTCTACCCATTCGGTGACT	
Chr14_9077210	F1:GAAGGTGACCAAGTTCATGCTTTAGGAATCTGGATGTACCCTATAAATTC**A**	A
	F2:GAAGGTCGGAGTCAACGGATTAGGAATCTGGATGTACCCTATAAATTC**C**	C
	R:GAAAATTGCTGCTCTTTTGGAACTAA	
Chr14_9077244	F1:GAAGGTGACCAAGTTCATGCTGAAAATTGCTGCTCTTTTGGAACTAA**T**	A
	F2:GAAGGTCGGAGTCAACGGATTGAAAATTGCTGCTCTTTTGGAACTAA**C**	G
	R:TCTGGATGTACCCTATAAATTCACGTT	
Chr14_9077247	F1:GAAGGTGACCAAGTTCATGCTGAAAATTGCTGCTCTTTTGGAAC**T**	A
	F2:GAAGGTCGGAGTCAACGGATTAAAATTGCTGCTCTTTTGGAAC**C**	G
	R:TCTGGATGTACCCTATAAATTCACGTT	
Chr17_9743860	F1:GAAGGTGACCAAGTTCATGCTAGTTATTATTGACTCACGGTGGCTATTTT**A**	A
	F2:GAAGGTCGGAGTCAACGGATTATTATTGACTCACGGTGGCTATTTT**G**	G
	R:CATCCATAGCAGCTACACTTCATCAC	
Chr05_9289732	F1:GAAGGTGACCAAGTTCATGCTAGTGCCACAAATCTTAGAACCATTAGAAT**C**	C
	F2:GAAGGTCGGAGTCAACGGATTGAGTGCCACAAATCTTAGAACCATTAGAAT**T**	T
	R:AAAACAAATTGTGGGTGTTTGAACCA	
Chr05_9292231	F1:GAAGGTGACCAAGTTCATGCTCCTTACTGAGCTTATTTACTGGTTTTCACTG**A**	A
	F2:GAAGGTCGGAGTCAACGGATTCTTACTGAGCTTATTTACTGGTTTTCACTG**G**	G
	R:CCATTTCATTCCCAATTCCTTAGTGG	
Chr05_9293973	F1:GAAGGTGACCAAGTTCATGCTTTTTTTAACCAATGTCATTCCATGCT**C**	C
	F2:GAAGGTCGGAGTCAACGGATTTTTTTAACCAATGTCATTCCATGCT**T**	T
	R:AAGGCCTTTAGTATCCATGGCTTTGC	
Chr05_11420622	F1:GAAGGTGACCAAGTTCATGCTACAGAAAACTCCATGTTTCGATGATT**C**	C
	F2:GAAGGTCGGAGTCAACGGATTACAGAAAACTCCATGTTTCGATGATT**T**	T
	R:CGGACTACTGTTGGTGGTCCTATTCG	
Chr05_11435508	F1:GAAGGTGACCAAGTTCATGCTAAGTGCTGAAAGAAAAGGTCAGTTTATG**A**	A
	F2:GAAGGTCGGAGTCAACGGATTGAAGTGCTGAAAGAAAAGGTCAGTTTATG**T**	T
	R:GGTGATGGTTTGAATTGATTGTCACA	

Chr05_12148738	F1:GAAGGTGACCAAGTTCATGCTCAACATCACCACCCCTTACTGAG**G**	G
	F2:GAAGGTCGGAGTCAACGGATTCAACATCACCACCCCTTACTGAG**A**	A
	R:TTATCGTGGTTGTGAAGGCAGTGATT	
Chr05_12157706	F1:GAAGGTGACCAAGTTCATGCTCACACTACGCTCTGGAATAAAGAACATTA**G**	C
	F2:GAAGGTCGGAGTCAACGGATTCACACTACGCTCTGGAATAAAGAACATTA**T**	A
	R:TGAGAGATTGAAAATACAAGTACAAAGCATGA	
Chr05_12160731	F1:GAAGGTGACCAAGTTCATGCTTGCATTTGTTCGGTTATTATCTACGAA**A**	A
	F2:GAAGGTCGGAGTCAACGGATTAATGCATTTGTTCGGTTATTATCTACGAA**T**	T
	R:CAATGTTGTGCTTAAGGAATTGTTGT	
Chr05_17106547	F1:GAAGGTGACCAAGTTCATGCTGGATGACGCTCTTAGTTAACGTGTGA**C**	C
	F2:GAAGGTCGGAGTCAACGGATTGGATGACGCTCTTAGTTAACGTGTGA**T**	T
	R:TCTGACTGAATGAGACATCACCTGAA	
Chr06_26871928	F1:GAAGGTGACCAAGTTCATGCTGTGTCTAAGCCTCTTTATATGATTTAAGCTTC**T**	T
	F2:GAAGGTCGGAGTCAACGGATTTGTCTAAGCCTCTTTATATGATTTAAGCTTC**G**	G
	R:ATGCCCCGGTAGCATACAAAAGATTT	
Chr13_1598790	F1:GAAGGTGACCAAGTTCATGCTTTCTTCTGGTAAAGTTCTGCTACCTGAT**G**	G
	F2:GAAGGTCGGAGTCAACGGATTCCTTCTTCTGGTAAAGTTCTGCTACCTGAT**A**	A
	R:ACTGCACCTGACTGGAAAAGATCACA	

Supplementary Table S8 KASP Reaction System

	**Final concentration**	**Actual usage**
100μM Primer C	0.42μM	0.0033μl
100μM Primer X	0.17μM	0.0013μl
100μM Primer Y	0.17μM	0.0013μl
2× KASP Master Mix	1×	0.3945μl
Ultrapure water		0.3995μl
DNA（dry）		20ng-50ng
Total volume		0.8μl

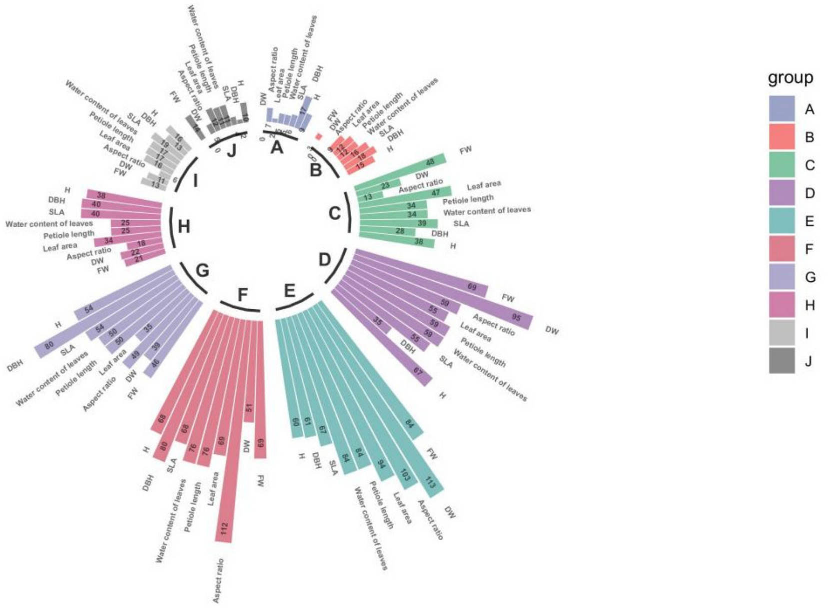

Supplementary Figure S1 Distribution of phenotypic traits in 10 grades of germplasm resources. A-J denotes 10 classes, and the height of the column indicates the distribution of the trait in this class.

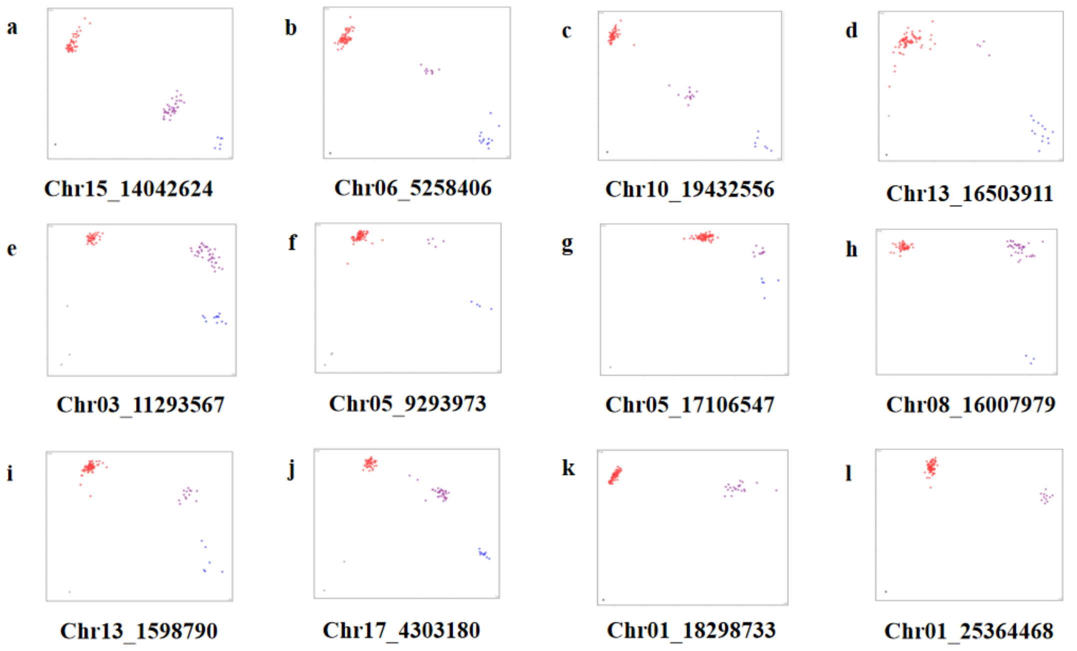

Supplementary Figure S2 Genotyping of 12 KASP markers. Class A typing indicates that the sample contains a pure A allele at this KASP marker locus (labeled red in the typing plot and located in the upper left corner of the graph), class B typing indicates that the sample contains a pure B allele at this KASP marker locus (labeled blue in the typing plot and located in the lower right corner of the graph), and heterozygous typing indicates that the sample contains an A- and B-heterozygous allele at this KASP marker locus ((labeled purple in the typing graph and located close to the 45 degree axis of the coordinate axis). Blank controls (NTC) and samples with no detectable genotypes (labeled gray in the typing graph, located near the origin of the coordinates).
